# Improving the Performance of Composite Hollow Fiber Membranes with Magnetic Field Generated Convection Application on pH Correction

**DOI:** 10.3390/membranes11060445

**Published:** 2021-06-15

**Authors:** Aurelia Cristina Nechifor, Alexandru Goran, Vlad-Alexandru Grosu, Constantin Bungău, Paul Constantin Albu, Alexandra Raluca Grosu, Ovidiu Oprea, Florentina Mihaela Păncescu, Gheorghe Nechifor

**Affiliations:** 1 Analytical Chemistry and Environmental Engineering Department, University Politehnica of Bucharest, 1-7 Polizu St., 011061 Bucharest, Romania; aureliacristinanechifor@gmail.com (A.C.N.); alexandru@santego.ro (A.G.); florynicorici@yahoo.com (F.M.P.); gheorghe.nechifor@upb.ro (G.N.); 2 Department of Electronic Technology and Reliability, Faculty of Electronics, Telecommunications and Information Technology, University Politehnica of Bucharest, Bd. Iuliu Maniu, nr. 1-3, 061071 București, Romania; 3 Department of Engineering and Management, Faculty of Management and Technological Engineering, University of Oradea, 410087 Oradea, Romania; bungau@uoradea.ro; 4 IFIN Horia Hulubei, Radioisotopes and Radiation Metrology Department (DRMR), 30 Reactorului St., 023465 Măgurele, Romania; paulalbu@gmail.com; 5 Department of Inorganic Chemistry, Physical Chemistry and Electrochemistry, University Politehnica of Bucharest, 1-7 Polizu St., 011061 Bucharest, Romania; ovidiu.oprea@upb.ro

**Keywords:** pH correction, aluminum retention, ethylene propylene diene monomer sulfonate, polypropylene hollow fibers, impregnated membranes, magnetic convection

## Abstract

The membranes and membrane processes have succeeded in the transition from major technological and biomedical applications to domestic applications: water recycling in washing machines, recycling of used cooking oil, recovery of gasoline vapors in the pumping stations or enrichment of air with oxygen. In this paper, the neutralization of condensation water and the retention of aluminum from thermal power plants is studied using ethylene propylene diene monomer sulfonated (EPDM-S) membranes containing magnetic particles impregnated in a microporous propylene hollow fiber (I-PPM) matrix. The obtained membranes were characterized from the morphological and structural points of view, using scanning electron microscopy (SEM), high resolution SEM (HR-SEM), energy dispersive spectroscopy analysis (EDAX) and thermal gravimetric analyzer. The process performances (flow, selectivity) were studied using a variable magnetic field generated by electric coils. The results show the possibility of correcting the pH and removing aluminum ions from the condensation water of heating plants, during a winter period, without the intervention of any operator for the maintenance of the process. The pH was raised from an acidic one (2–4), to a slightly basic one (8–8.5), and the concentration of aluminum ions was lowered to the level allowed for discharge. Magnetic convection of the permeation module improves the pH correction process, but especially prevents the deposition of aluminum hydroxide on hollow fibers membranes.

## 1. Introduction

**Figure 1:**
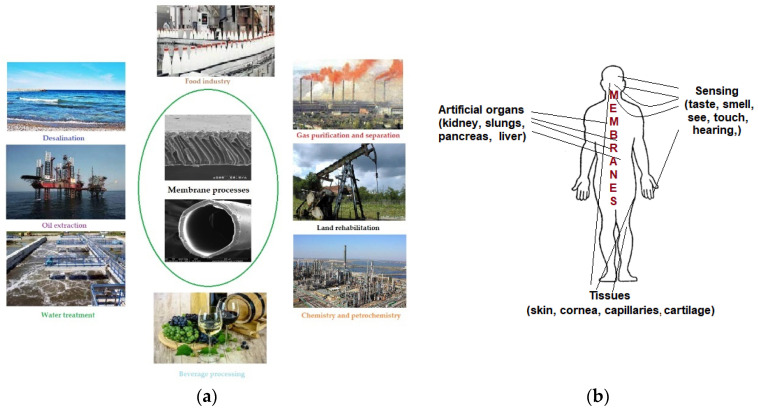
Membrane applications (**a**) technological applications: desalination, oil extraction, water treatment, food industry, beverage processing, gas purification and separation, land rehabilitation, chemistry and petro–chemistry; (**b**) biomedical applications: artificial organs (kidneys, pancreas, lungs, liver); sensing (taste, smell, hearing, touch, see); tissues (skin, cornea, veins, capillaries, arteries, cartilage).

Membrane technology has known consecration through two very successful applications, in two different fields of human activity [1,2]. The first is a large-scale and high-impact technological application referring to obtaining drinking water by the desalination of sea water [3,4], and the second is an exceptional biomedical achievement: the artificial kidney [5,6] (Figure 1).

Starting from these landmarks, membranes and membrane processes have succeeded due to flow performance, low costs with chemical reagents and the easy scaling up the development of remarkable industrial applications, such as the: separation of petroleum products, membrane electrolysis of sodium chloride, gases separation and purification, industrial water treatment, protein separation and concentration and the purification, separation and sterilization of food fluids (Figure 1a) [7,8,9,10,11,12,13,14]. At the same time, the special selectivity and the possibility of adjusting the sensitivity through various operational parameters (pH, ionic strength, redox potential, reactant concentration, volatility, electrical or magnetic properties) determined a multitude of biomedical applications, such as: tissue grafts, controlled drug release, an artificial lung, an artificial pancreas and, recently, the simulation of liver function (Figure 1b) [15,16,17].

Lately, even the domestic applications of membranes have multiplied, some examples worth mentioning are the recycling of water in washing machines and car washes, the recycling of food and car manufacturing industry oils, air filtration and/or its concentrations of oxygen or nitrogen, the recovery of vapors from gas stations, or at correction of the unpleasant smell of the premises [18,19,20,21,22]. 

All of the applications presented are based on an extraordinary diversification of membranes, which are based on the special development of polymeric, oxide, metallic and composite materials, but especially on the appearance of nanometric shapes with predetermined dimensions and properties [23,24,25,26].

Nanometric dimensions and nano-structuring have deeply penetrated the development of membranes that are symmetrical and asymmetrical, composite or mixed, liquid or on support [27,28].

The design of membranes has constantly evolved from flat to hollow fiber, and the processing mode (continuous, batch, stepped) has led to the increased operating performance of various membranes [29,30].

All of these advances in membrane technology have allowed the approach of more and more specific applications, either in terms of the separate chemical species or in terms of the matrix from which these chemical species are separated [31,32,33,34].

Among the technical problems of the membrane processes in operation, the most important are the clogging or fouling of the membranes and the polarization concentration, which lead to decreased flow over time and sometimes to decreased membrane life [35,36,37,38]. Biofouling is also considered in the case of systems that allow microbiological activity [39,40]. These aspects that appear in the membrane processes are long studied and countless solutions have been found, such as: regulating the flow regimes, alternative flow through modules, system vibration, cyclic operation-washing cycles and the use of ultrasound [41,42,43,44,45]. In the case of biofouling the most commonly used solution is the introduction of silver nanoparticles into membranes or into a separate system [46,47]. These multiple solutions show that the problem is not yet fully solved, especially in particular cases where the flow regime cannot be imposed [48,49,50,51,52].

Such an application is related to the correction of pH and the water composition from the medium-capacity heating plants, dedicated to homes, hospitals, schools, administrative or office buildings [51]. 

Condensing heating plants, the only ones currently accepted for the production of gas energy in cities, produce condensation water, which in European Union countries is no longer allowed to be discharged into sewage water, both because they have a low pH and because of the content of metal ions [21,51].

Aspects such as acid pH and metal ion content or anions can be successfully solved using membranes and membrane processes. Water treatment using membrane techniques has solved a large part of the problem regarding effluents discharged in small quantities, with a pH close to the limits allowed for discharge or containing traces of metal ions.

This paper addresses the correction of pH and the elimination of aluminum ions from condensation waters with a membrane system based on a microporous membrane polypropylene hollow fiber (PPM), impregnated with ethylene propylene diene monomer sulfonate (EPDM-S) and magnetic particles, operated in variable magnetic field.

## 2. Materials and Methods

### 2.1. Materials

#### 2.1.1. Chemicals

The materials used in the present work were of analytical purity. The following were purchased from Merck (Merck KGaA, Darmstadt, Germany): aluminum sulfate (Al_2_SO_4_), iron wire (Fe), silver nitrate (AgNO_3_), sodium hydroxide (NaOH) and hydrochloric acid solution (HCl, 35%), and were used without further purification. 

The purified water, characterized by 18.2 µS/cm conductivity, was obtained with a RO Millipore system (MilliQR Direct 8 RO Water Purification System, Merck, Darmstadt, Germany).

The ethylene propylene diene monomer sulfonate (EPDM-S) 30% toluene solution had a 300 µmol-g ion capacity exchange (ICE) [53].

#### 2.1.2. Membrane Support

**Figure 2:**
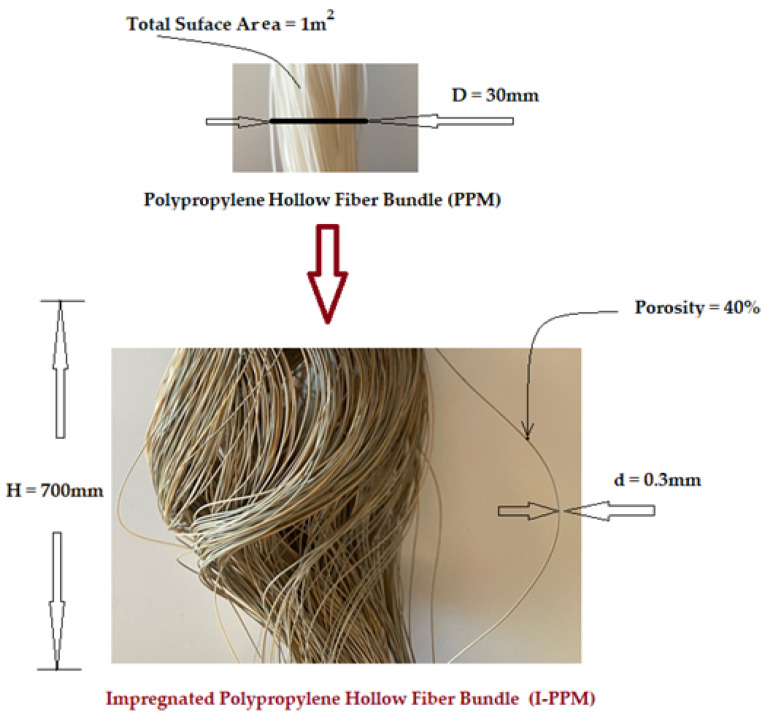
The characteristics of the hollow fibers polypropylene support membranes (PPM) and impregnated polypropylene hollow fiber membrane (I–PPM).

The hollow fibers polypropylene support membranes (PPM) were provided by GOST Ltd., Perugia, Italy. Their principal characteristics are presented in Figure 2 [18,21,22]. 

### 2.2. Impregnated Ethylene Propylene Diene Monomer Sulfonate (EPDM-S), Magnetic Nanoparticles (MNp) Hollow Fiber Membrane Preparation (I-PPM)

#### 2.2.1. Obtaining the Magnetic Nanoparticles

The magnetic nanoparticles proposed for use in these experiments were obtained using a known electrochemical method [54], presented in detail in [22,54]. In short, the method consists of an electrolysis process in a cell with an iron anode and cathode and a silver nitrogen electrolyte, using cyclic voltammetry technique. The nanoparticles used in this study were dimensionally and morphologically characterized using scanning electron microscopy (SEM), energy dispersive spectroscopy analysis (EDAX) as well as from the perspective of saturation magnetization.

#### 2.2.2. Obtaining the Dispersion of Magnetic Nanoparticles (MNp) in EPDM-S Solution in Toluene

The dispersion of magnetic nanoparticles (MNp) is achieved by introducing 100 mL of an EPDM-S solution (30% *w*/*w*), 900 mL of toluene and 20 g of MNp into a conical glass bottle with a wheeled plug. Then, the bottle is stirred in an ultrasonic bath for at least 4 h, until the formation of a homogeneous-looking dispersion is observed. 

#### 2.2.3. Impregnation of Hollow Fibers Polypropylene Membranes with Magnetic Dispersion 

The desired dispersion quantity is introduced in a graded cylinder and the polypropylene hollow fibers bundle is completely immersed (in a “U” shape) in the cylinder, having the connector ends outside. The connector ends are connected to a vacuum installation provided with a buffer vessel, for toluene capture, and the desired amount of dispersion is aspirated so as to impregnate the membrane (I-PPM). Samples from the impregnated membranes were analyzed using SEM, EDAX and thermal analysis. 

### 2.3. pH Correction Tests and Aluminum Ions Retention from Condensation Water 

#### 2.3.1. The Permeation Installation with I-PPM

**Figure 3:**
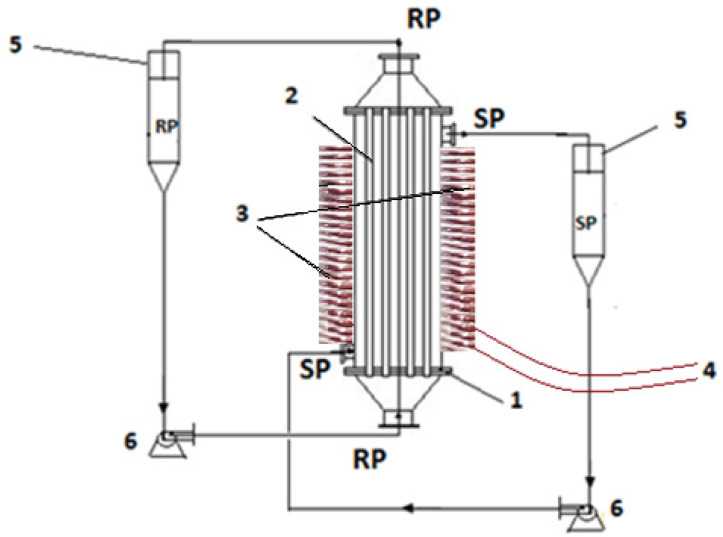
Schematic presentation of the pertraction module and its principal components: 1—pertraction module, 2—hollow fiber membrane, 3—electric bobbins, 4—oscillatory system, 5—reservoirs: SP—source phase, RP—receiving phase, 6—pumps.

Figure 3 shows the permeation installation in variable magnetic field, previously presented in detail [54], but this time the generating element is a set of electric coils.

The magnetic induction of a coil is imposed based on the following relation (1) at a value similar to that given by the ferrite used in a previous work:

*B* = *µnI/l* (1)

where: *B* is magnetic induction; *µ* is environmental permissiveness, *n* is the number of turns, *I* is electric field intensity, and *l* is the coil length.

On to the permeation module were placed six coils with the alternating inductance vector (up–down) made by reversing the direction of the electric current through the coils.

#### 2.3.2. Carrying out the Tests in the Permeation Installation

The transport of protons and aluminum ions was performed using the above described permeation installation that allows the circulation of the source phase outside the membrane bundle and the receiving phase inside the hollow fibers (Figure 3). There is a 10 liter tank for the supply solution and a 2 liter tank for the receiving solution. The electric coils placed outside the permeator will induce a variable field.

The fluxes from the source phase [55,56] were determined against the measured permeate mass within a determined time range, applying the following equation:

(2) $$J=\frac{M}{S\times t}\left(g/m^{2}h\right)$$

where: *M* = the permeate mass (g), *S* = the effective surface of the membrane (m^2^) and *t* = the time necessary to collect the permeate volume (h).

The extraction efficiency (EE%) of the analytes calculated using the concentration or absorbance of the solutions [57,58] are, respectively, as follows:

(3) $$EE\left(\%\right)=\frac{\left(c_{0}-c_{f}\right)}{c_{0}}\times 100$$

where *c_f_* is the final concentration of the solute (aluminum) and *c*_0_ is the initial concentration of solute (aluminum).

(4) $$EE\left(\%\right)=\frac{\left(A_{0}-A_{s}\right)\;}{A_{0}}\;\times 100$$

where *A*_0_ is the initial sample solution absorbance and *A_s_* is the current sample absorbance.

### 2.4. Equipment 

The microscopy studies, SEM and HFSEM, were performed on a Hitachi S4500 system (Hitachi High-Technologies Europe GmbH, Mannheim, Germany). Thermal characterizations were performed on a Netzsch Thermal Analyzer (NETZSCH-Gerätebau GmbH, Selb, Germany). The thermal analysis took place in a nitrogen atmosphere at a 10 °C/min heating rate, from room temperature (25 °C) up to 900 °C.

The UV-Vis analyses of the aqueous aluminium solutions were performed on a Spectrometer CamSpec M550 (Spectronic CamSpec Ltd., Leeds, UK).

The electrochemical processes were followed up with a PARSTAT 2273 Potentiostat (Princeton Applied Research, AMETEK Inc., Oak Ridge, TN, USA). It has been used a glass cell with three electrodes setup.

XRD analyses were recorded using PANalytical X’Pert Pro MPD equipment (PANalytical B.V., Almelo, The Netherlands) with a CuKα radiation source, and a 2θ measurement range from 10 to 90 °C.

The nanoparticle magnetization diagrams were determined with a Quantum Design MPMS 3 Magnetometer (Quantum Design Europe, Darmstadt, Germany) based on superconducting quantum interference device detection (SQUID). The DC operation mode applied allowed us to run SQUID-vibrating-sample magnetometer (VSM) measurements.

The UV–Vis studies on the nanoparticles samples were performed on dual-beam UV equipment, Varian Cary 50 (Agilent Technologies Inc., Santa Clara, CA, USA), at a resolution of 1 nm, with a spectral bandwidth of 1.5 nm, and a 300 nm/s scan rate. The samples’ UV–Vis spectra were recorded for a wavelength from 200 to 800 nm, at room temperature, using 10 mm quartz cells.

To assess and validate the content in metal ions, the AAnalyst 400 AA Spectrometer (Perkin Elmer Inc., Waltham, MA, USA) with WinLab32 AA software (Perkin Elmer) atomic absorption spectrometer was used, with a single-element hollow-cathode lamp. 

## 3. Results and Discussion

The condensate generated by thermal power plants with an average power 50–150 KWh, is apparently clean water, but in reality, it has a pH between 2 and 4, which is quite aggressive, because the range allowed by the European Directive for water discharged directly into the canal is limited to ≥6.5; ≤8.5 [21,58,59,60,61,62].

For the removal of micro-pollutants from water and air or the recuperative separation of some substances, a determinant of the success of the chosen process is the type and performance of the membranes [63,64].

Using composite membranes, complex processes were performed, such as: colloidal ultrafiltration, pervaporation, pertraction, electro dialysis and liquid membranes [65,66,67,68]. All were evaluated in terms of process performance for solving domestic problems.

### 3.1. Impregnated Ethylene Propylene Diene Monomer Sulfonate—Magnetic Nanoparticles—Hollow Fiber Membrane (I-PPM)

The impregnated membranes used in this paper have the following three elements: polypropylene hollow fiber, magnetic nanoparticles and ethylene propylene diene monomer sulphonate.

**Figure 4:**
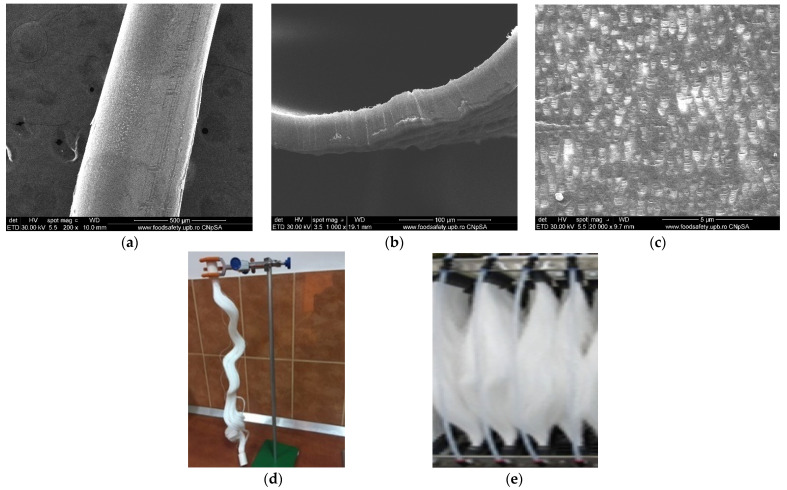
Scanning electron microscopy images of the support of impregnated membranes: (**a**) individual fiber; (**b**) fiber section; (**c**) pores details; photographic images of: (**d**) one hollow fiber bundle and (**e**) multiple hollow fiber bundles.

The hollow fiber membrane support matrix solves the following three important issues required by the membrane process [22,52,54]:

- porosity and large contact surface (Figure 2 and Figure 4a,b,c);
- physical chemical resistance: the entire pH range, temperatures up to 150 °C, insolubility in the usual solvents used for obtaining membranes;
- assembly and scaling possibilities: the fibers can be assembled in packages (bundles), which can be assembled in individual or multiple modules (Figure 2 and Figure 4d,e).

**Figure 5:**
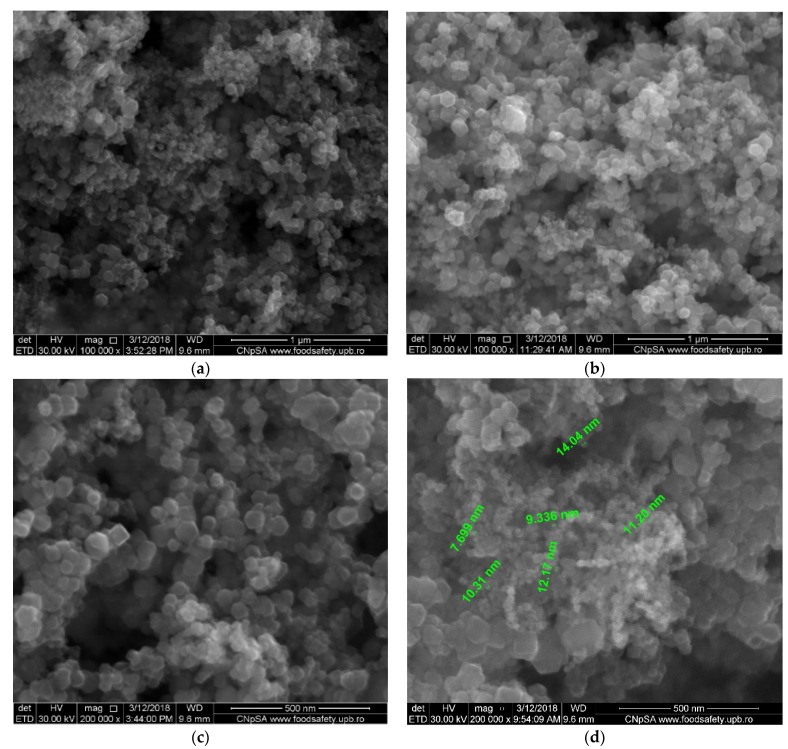

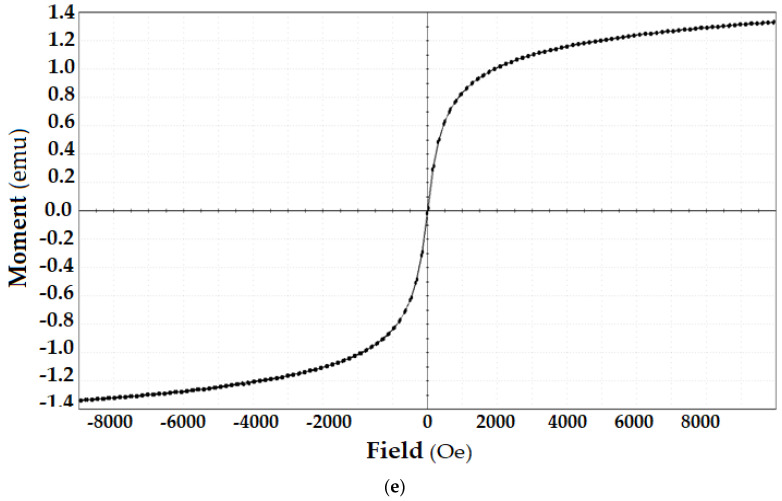
Scanning electron microscopy images of magnetic nanoparticles: (**a**,**b**) scanning electron microscopy for the nanoparticles; (**c**) detail of nanoparticles from (**a**); (**d**) specific dimensions of nanoparticles; (**e**) magnetization curve.

The magnetic nanoparticles required for impregnation were obtained using the electrochemical process [54] and are characterized by (Figure 5) the following:

- dimensions between 10 and 40 nm (Figure 5a);
- a relatively uniform distribution, compatible with polypropylene support (Figure 5b);
- a saturation magnetization of 1.334 emu.

The magnetization curve for the obtained nanoparticles is shown in Figure 5e. It was made at room temperature using a magnetometer that measures vibrations and shows that the sample has a degree of magnetic saturation of 1.334 emu. At the same time, the magnetization curve verifies that the nanoparticles are superparamagnetic, have zero magnetization and a coercivity of 10.960 Oe.

**Figure 6:**
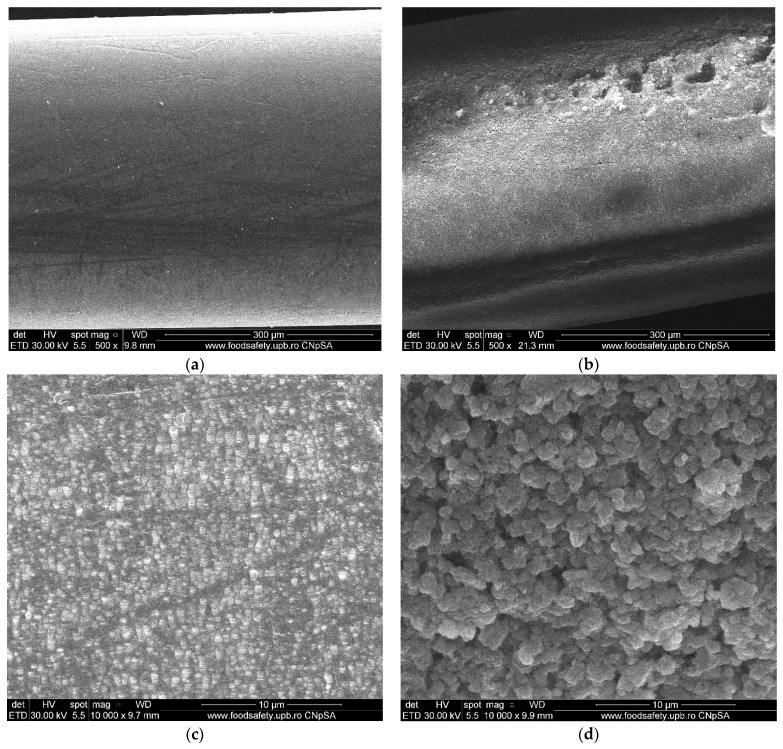
Scanning electron microscopy images of an impregnated ethylene propylene diene monomer sulfonate—magnetic nanoparticles—hollow fiber membrane (I–PPM): (**a**) PPM membrane surface; (**b**) I–PPM membrane surface of the right fiber; (**c**) PPM surface detail; (**d**) I–PPM surface detail.

The nanoparticles dispersed in a toluene sulfonated ethylene propylene diene monomer solution were impregnated on the polypropylene support, leading to composite membranes whose appearance is highlighted using scanning electron microscopy (Figure 6). On the right side of the image is the same fiber that has been uniformly coated and carefully processed for scanning electron microscopy in order to avoid exfoliation (Figure 6b). A relevant view of the two membranes is shown in Figure 6c,d. Figure 6c shows the membrane surface before coating or dispersion, to reveal the distribution and uniformity of the pores on the surface. Figure 6d shows the image of the dispersion coated fiber, observing the agglomerations of the nanoparticles coated with sulfonated ethylene propylene diene monomer.

**Figure 7:**
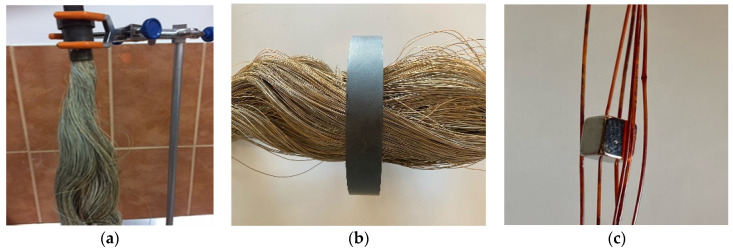
Images of an impregnated membrane (I–PPM) (**a**) and an illustration of magnetic properties (**b**,**c**).

The microporous support pores (Figure 4c and Figure 6c) were coated with polymeric dispersion [22,54], obtaining an ion exchange membrane with magnetic properties (Figure 6d and Figure 7).

**Figure 8:**
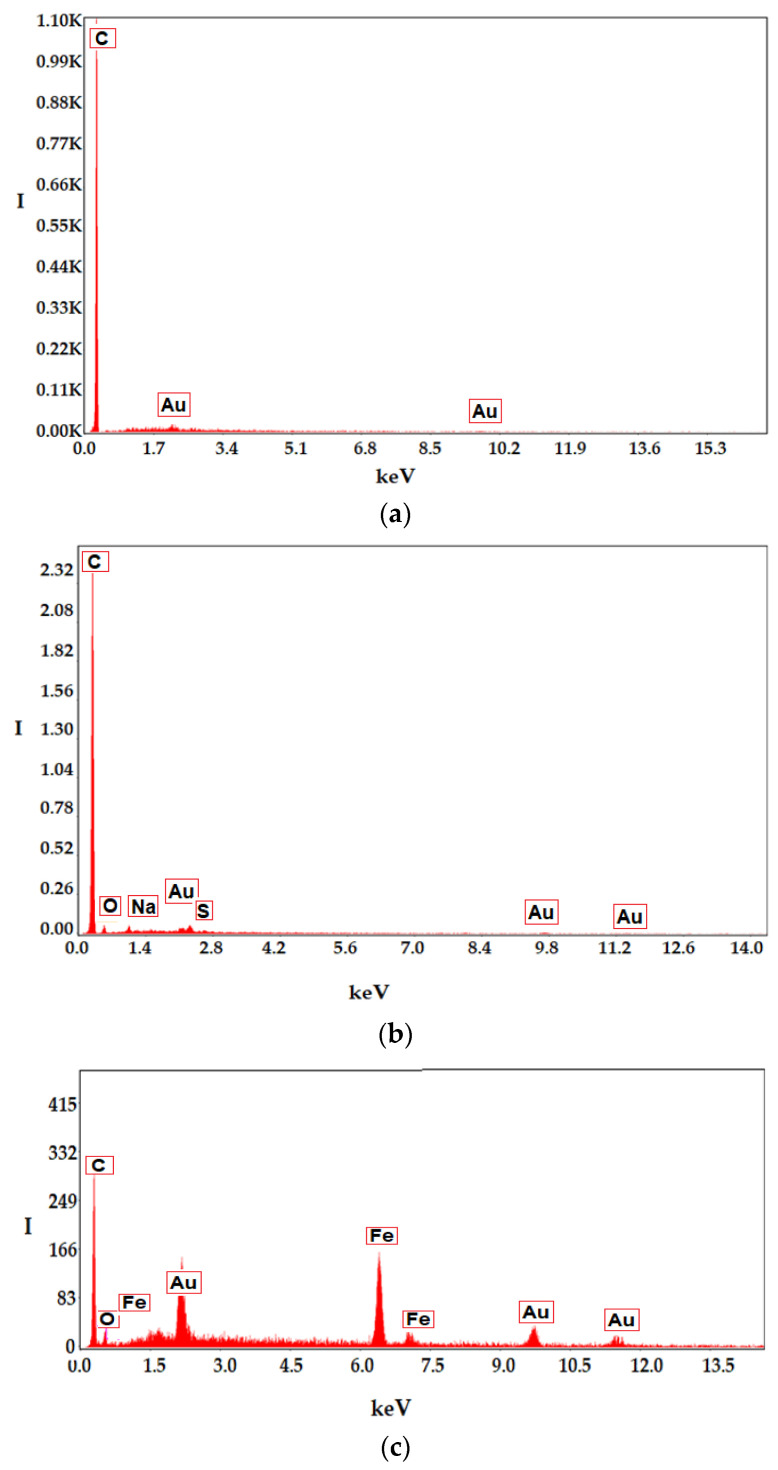
Analysis using energy dispersive spectroscopy (EDAX) of the membrane before coating (**a**); and polymer coated membrane (**b**); respectively, of the membrane coated with nanodispertion of magnetic particles (**c**).

The energy dispersive spectroscopy (EDAX) analysis of the membrane before coating and of the membrane covered after processing in the district heating installation (Figure 8) indicates the superficial composition of the samples. Thus, the support membrane is composed only of carbon and hydrogen (polypropylene—Figure 8a), and the impregnated membranes are composed either of carbon, oxygen, sulfur, sodium and hydrogen (Figure 8b) or of carbon, oxygen, iron and hydrogen (Figure 8c).

**Figure 9:**
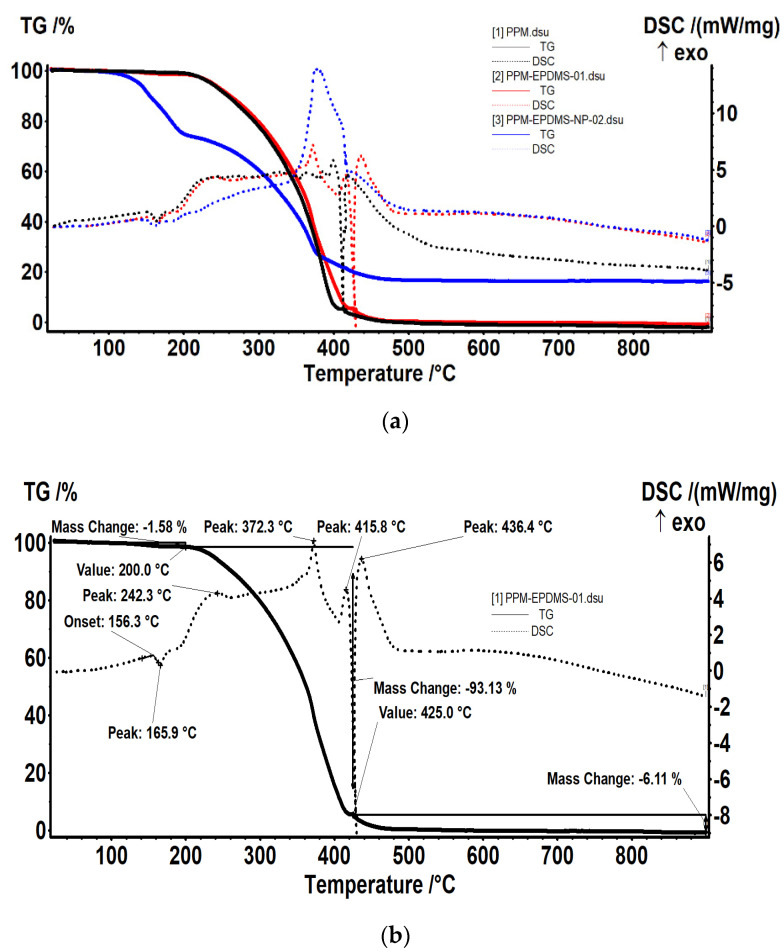

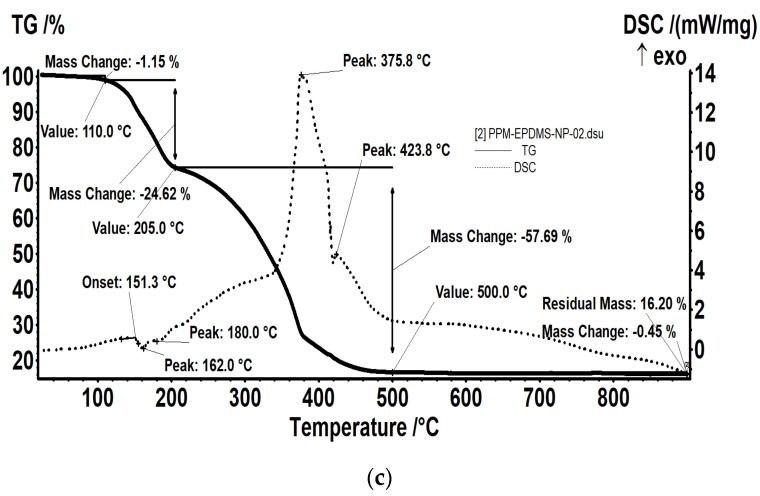
Thermal analysis of the membranes: (**a**) comparative thermal analysis hollow fiber membrane (PPM), EPDM–S impregnated hollow fiber membrane (EPDMS–PPM), and nanoparticle EPDMS hollow fiber membrane (PPM–EPDMS–NP); (**b**) PPM–EPDMS discussion diagram; (**c**) PPM–EPDMS–NP discussion diagram.

The thermal analysis is presented in Figure 9. The sample PPM–EPDMS (Figure 9b) loses 1.58% of initial mass in the RT-200 °C temperature interval, probably because of some adsorbed solvent molecules and plasticizers. In this interval, a small endothermic effect can be observed on the DSC curve, with the onset at 156.3 °C and the peak at 165.9 °C, which corresponds to the melting of polypropylene.

In the 200–425 °C temperature interval the main degradation process occurs, with the recorded mass loss being 93.13%. The process is accompanied by a series of exothermic effects on DSC curve, which are partially overlapped, with maximums at 242.3, 372.3 and 415.8 °C. This indicates that the sample undergoes a series of oxidative–degradative reactions. The carbonaceous residual mass is burned away at 425 °C, when a mass loss of 6.11% is recorded. On the DSC curve, a strong exothermic effect is present, with a maximum at 436.4 °C.

The sample PPM–EPDMS–NP (Figure 9c) loses 1.15% of initial mass in the RT-110 °C temperature interval, probably due to some adsorbed solvent molecules. Between 110–205 °C, the sample loses 24.62% of its initial mass. In this interval a small endothermic effect can be observed on the DSC curve, with the onset at 151.3 °C and the peak at 162.0 °C, which corresponds to the melting of polypropylene. At 180 °C, there is also a small shoulder on the endothermic peak, indicating the existence of a decomposition process.

In the 205–500 °C temperature interval the main degradation process occurs, the recorded mass loss being 57.69%. On the DSC curve, the process is accompanied by a series of exothermic effects, which are partially overlapped. Over the broad, asymmetric base exothermic effect at least two intense peaks are visible, with maximums at 375.8 and 423.8 °C. Both effects are asymmetric, with shoulders indicating a complex oxidative process. The carbonaceous residual mass is burned away, most probably after 400 °C, with the effect of 423.8 °C being assigned to this process. The small exothermic peaks at 555.7 and 573.4 °C can be assigned to the transformation of maghemite to hematite. The residual mass is red, formed by hematite, and has a value of 16.20%.

### 3.2. pH Correction and Aluminum Ions Retention Tests with Magnetic Ion Exchange Hollow Fiber Impregnated Membrane

#### 3.2.1. Operation of the Permeation Module in Magnetic Field

The initial tests for the ion-exchange magnetic fiber module were performed in order to establish the frequency of the oscillations of the electric current passing through the coils of the permeation installation to correct the pH of the synthetic aqueous solution.

**Figure 10:**
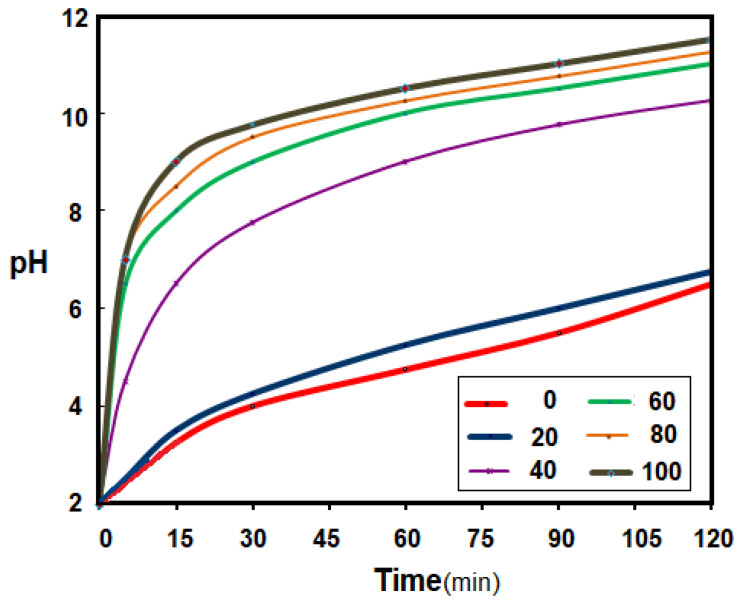
The evolution of the pH depending on the frequency of the electric current oscillations through the coils of pertraction installation: the numbers of oscillations are 0, 20, 40, 60, 80, 100 osc/min.

The evolution of the pH of the source phase was followed (Figure 10) depending on the frequency of current oscillations through the electrical coils. The value of the electric current intensity through the coils was chosen to achieve the magnetic field necessary for the interaction with the fibers (*i* = 1 A), the number of coils was fixed at six and the number of turns on each coil was *n* = 50 and the length of the coil was *l* = 10 cm. The distance between two successive coils was 5 cm. The electric current flows through the coils alternately from right to left and then from left to right, to ensure that the fibers acquire an individualized peristaltic movement. The direct current switch ensures the variation of 20, 40, 60, 80 and 100 oscillations per minute. The synthetic source solution, which circulates outside the impregnated membranes, has a pH of 2, provided by a 0.01 mol/L hydrochloric acid solution. The receiving solution, a solution of sodium hydroxide 1.0 mol/L, circulates inside the impregnated membranes and ensures the pH correction.

The use of the membrane module to correct the pH of the thermal power plant condensate has proved to be useful and efficient [21,55], but the increase and/or maintenance of the process performances during a long operation requires the preservation of the membrane’s characteristics. In order to avoid clogging and polarization by concentration, the use of the supply flow regime is not possible because the condensate flow is small and variable. This is why the option of obtaining convection using magnetic stirring was chosen.

The use of a variable magnetic field to increase the performance of protein ultrafiltration or nitrophenol pertraction has recently been reported [54]. In this study, a magnetic field generated by electric coils is used to agitate the fibers, which is much simpler and more precise to drive.

By varying the direction of the electric current through the coils by a predetermined number using an electric switch, relevant results were obtained that allowed a choice of the number of oscillations per minute of the current (Figure 10). The parameter followed was the pH, because it is easy to follow using well-established and precise electrochemical techniques. It has been found that 20 oscillations per minute improves the pH correction regime, but efficiency and rapidity are obtained at over 40 oscillations per minute. After 80 oscillations per minute, the efficiency increase is capped, with a value of 80 oscillations per minute being appropriate for subsequent experiments.

It is remarkable that in all cases, the large contact surface and the membrane composition ensures the main pH correction in the first 20 min of operation.

#### 3.2.2. pH Correction and Removal of Aluminum Ions in the Pilot Experiment

**Figure 11:**
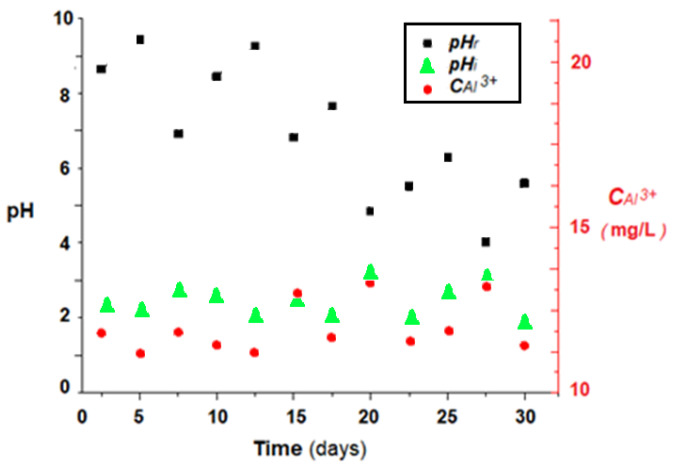
Evolution of the pH and aluminum ions concentration of a condensate of a thermal power plant in condensation during a winter month: pH_i_ in power plant effluent; pH_r_ in condensate tank; *C*_Al_^3+^ aluminum ions concentration in effluent.

In order to follow the efficiency of pH correction and aluminum ions concentration reduction, the permeation module was connected to the condensate evacuation of a heating plant of 50 KWh, for which the working temperature of the thermal agent was adjusted to 50 °C. Normally, such an installation produces 1–5 L/h of condensation, depending on the outside temperature that determines the combustion regime of the boiler. Figure 11 shows the pH and condensation parameters in the condensate tank.

The pH of the condensate (Figure 11) varies between two and three units, being most likely influenced by the combustion regime of the natural gas, but also by its composition. The concentration of aluminum ions in the condensate varies between 12 and 14 mg/L, but in previous studies it was found that it can reach 20–25 mg/L, being most often influenced by the pH of the condensate, which, when decreases below value of 2, causes significant corrosion of the aluminum elements of the thermal power plant.

The pH correction experiments, and the removal of aluminum ions were performed by coupling the permeation module to the thermal power plant exhaust to ensure the condensate flows through the outside of the membranes, and operated without magnetic stirring or with 80 oscillations per minute, of 1 A current, through the six alternating electric coils. The receiving phase, which circulates through the impregnated membranes, was placed in a tank of 2.0 L 1M sodium hydroxide solution, which ensures the circulation by free fall.

**Figure 12:**
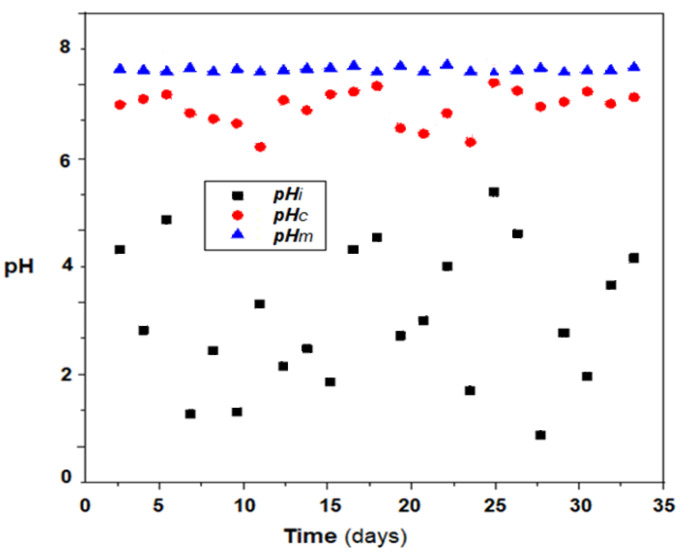
Evolution of the pH of a condensate of a thermal power plant in condensation during a winter month: pH_i_, in power plant effluent; pH_c_, by membrane treatment without magnetic stirring; pH_m_, after treatment with the magnetic stirring system.

**Figure 13:**
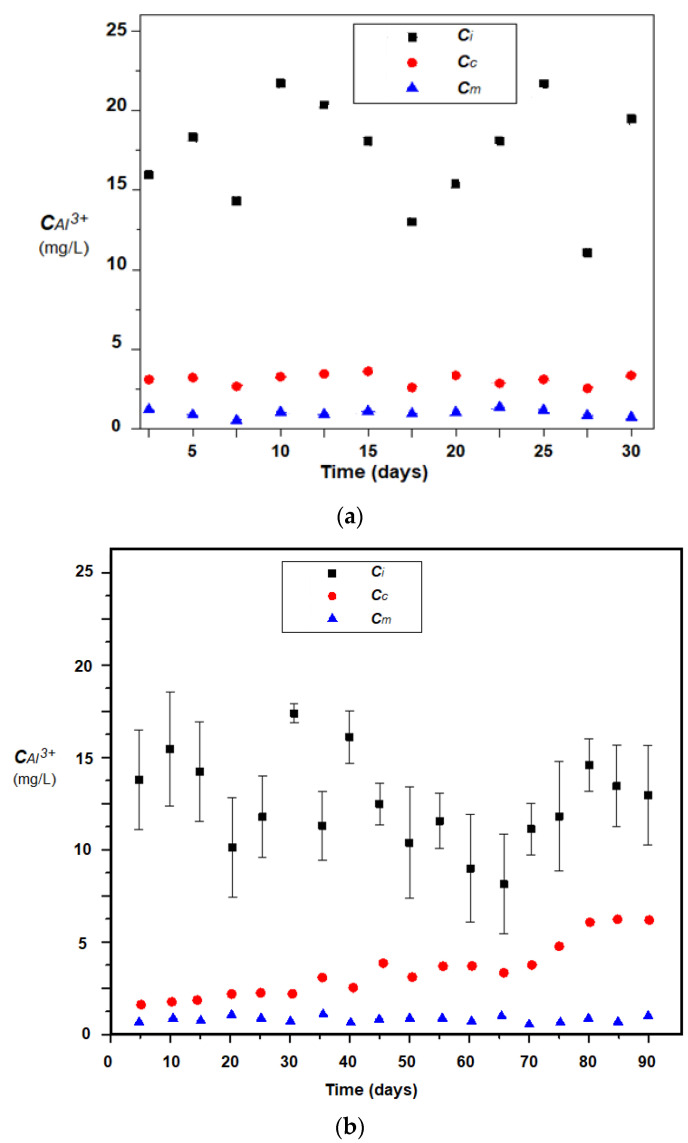
Evolution of aluminum ions concentration in the condensate of a condensing thermal power plant during: (**a**) a single winter month; (**b**) three winter months; C_i_—the condensate concentration evolution without treatment; C_c_—evolution of concentration by membrane treatment; C_m_—evolution of concentration by treatment with magnetically agitated membranes.

The results obtained in the pilot operation in a winter month indicate a correction of the pH (Figure 12) and a reduction in the load with aluminum ions (Figure 13a,b), both using the module without magnetic stirring, and using magnetic convection generation. However, it is remarkable to keep the pH relatively constant when generating magnetic convection, regardless of the pH of the boiler condensate. In the case of aluminum removal, the magnetic convection is much more efficient, a fact confirmed for a three-month operation (Figure 13b). 

**Figure 14:**
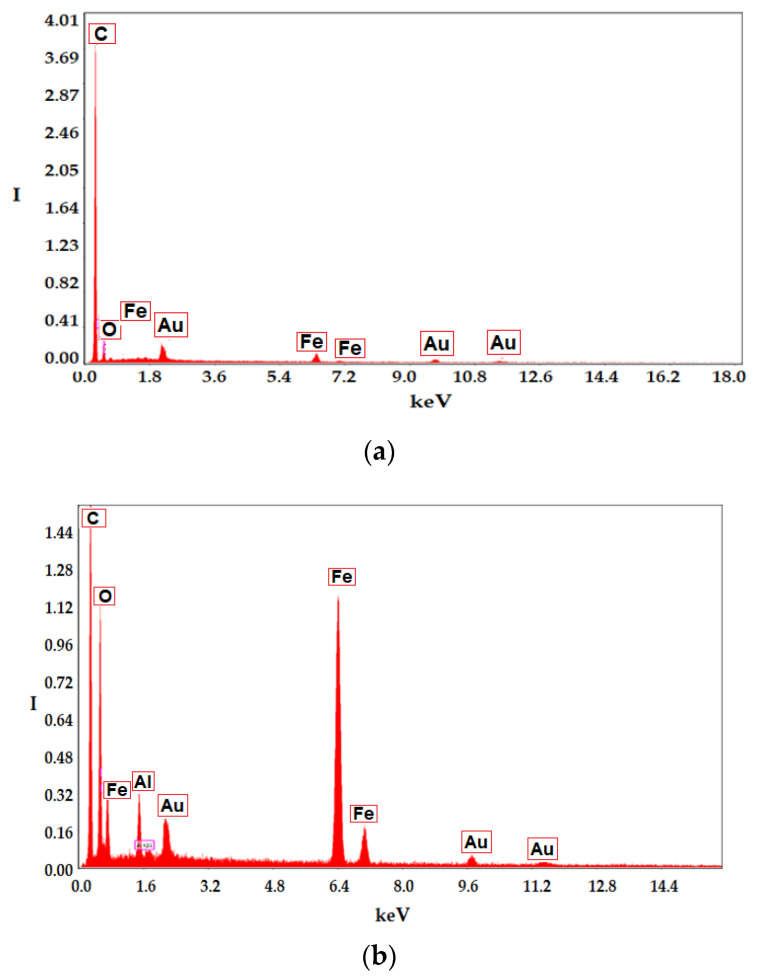
EDAX on the surface of the membranes in the module: (**a**) with magnetic stirring; (**b**) without magnetic stirring.

It is observed (Figure 13b) that if the membrane module is not provided with efficient magnetic stirring, then the removal of aluminum ions decreases constantly, while in the case of magnetic convection the removal of aluminum ions is better and is maintained throughout the studied interval. This behavior is most likely caused by the accumulation of aluminum hydroxide on the surface of the impregnated membrane. According to the surface analysis (EDAX-Figure 14) of the membrane samples taken after the operating interval, it turns out that in the case of the module without magnetic convection, the membranes only contain the initial constituent elements (Figure 14a), while the membranes of the module without magnetic stirring also contain aluminum.

In order to avoid a possible clogging by biofouling, the magnetic nanoparticles that enter in membrane composition (I-PPM) have a quantity of silver, coming from the electrochemical preparation procedure of the magnetic nanoparticles in the presence of electrolyte silver nitrate solution, which was recently reported [54].

### 3.3. The Mechanism of Convective Transport Generated by the Oscillating Magnetic Field

The obtained impregnated membranes (I-PPM) ensure, for the chosen application, both the correction of the pH and the removal of aluminum ions from the condensate of the thermal power plants, and meet the following conditions:

- large contact surface;
- physical-chemical resistance over the entire pH range;
- relatively fast ion exchange;
- avoiding biofouling;
- the possibility of improving mass transfer using magnetically induced convection.

**Figure 15:**
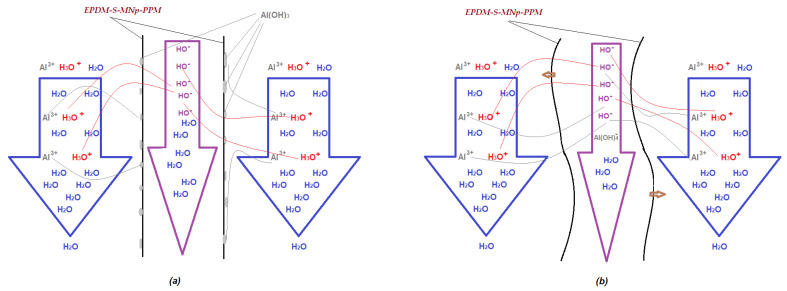
Schematic representation of the interactions in transfer through impregnated membranes: (**a**) without magnetic stirring; (**b**) with magnetic stirring (little brown arrows).

From the point of view of the mass transfer mechanism through the membrane system (Figure 13):

- mass transfer is generated by the concentration gradient (pH and aluminum ions concentration);
- the maintenance of the material flow is ensured by the neutralization reaction and respectively the complexation of the aluminum ions is ensured with hydroxyl ions;
- convection is improved by magnetic stirring because the supply flow with the condensate is small and conditioned by the power plant’s operation;
- the clogging of the non-magnetically stirred membranes is determined by the formation of aluminum hydroxide (Figure 15a).

Although the membrane system without agitation is quite efficient (Figure 10, Figure 11, Figure 12 and Figure 13), ensuring a constant pH correction and removal of aluminum ions is significantly improved by generating magnetic convection.

## 4. Conclusions

In this paper we have presented the results of the pH correction and the retention of aluminum ions from the condensate of medium power heating plants.

The pH correction and the retention of aluminum ions are based on the use of membranes impregnated with ethylene propylene diene monomer sulfonate (EPDM-S) containing magnetic particles impregnated in a microporous matrix of propylene hollow fiber (PP-HF) by operating in a magnetic field generated by electric coils.

The obtained membranes were characterized both morphologically and in terms of process performance, and were effective without the magnetic stirring of the membranes, especially when generating a magnetic convection.

Improving the process of the pH correction and the retention of aluminum ions for condensing water in medium power heating plants consists of faster neutralization and a more advanced removal of aluminum.

Avoiding the clogging of the membrane with aluminum hydroxide was highlighted by analyzing the surface of the membranes after use.

The pH was raised from an acidic one (2–4) to a slightly basic one (8–8.5) and the concentration of the aluminum ions was lowered to the level allowed for discharge. Magnetic convection of the permeation module improves the pH correction process, but especially prevents the deposition of aluminum hydroxide on hollow fiber membranes.
